# Machine learning using clinical data at baseline predicts the medium-term efficacy of ustekinumab in patients with ulcerative colitis

**DOI:** 10.1038/s41598-024-55126-1

**Published:** 2024-02-22

**Authors:** Hiromu Morikubo, Ryuta Tojima, Tsubasa Maeda, Katsuyoshi Matsuoka, Minoru Matsuura, Jun Miyoshi, Satoshi Tamura, Tadakazu Hisamatsu

**Affiliations:** 1 Department of Gastroenterology and Hepatology, Kyorin University School of Medicine, 6-20-2 Shinkawa, Mitaka-shi, Tokyo 181-8611 Japan; 2 Department of Electrical, Electronic and Computer Engineering, Faculty of Engineering, Gifu University, Gifu, Japan; 3 Division of Gastroenterology and Hepatology, Department of Internal Medicine, Toho University Sakura Medical Center, Chiba, Japan

**Keywords:** Ulcerative colitis, Machine learning, Ustekinumab, Steroid-free clinical remission, Prediction model, Gastroenterology, Gastrointestinal diseases

## Abstract

Predicting the therapeutic response to biologics before administration is a key clinical challenge in ulcerative colitis (UC). We previously reported a model for predicting the efficacy of vedolizumab (VDZ) for UC using a machine-learning approach. Ustekinumab (UST) is now available for treating UC, but no model for predicting its efficacy has been developed. When applied to patients with UC treated with UST, our VDZ prediction model showed positive predictive value (PPV) of 56.3% and negative predictive value (NPV) of 62.5%. Given this limited predictive ability, we aimed to develop a UST-specific prediction model with clinical features at baseline including background factors, clinical and endoscopic activity, and blood test results, as we did for the VDZ prediction model. The top 10 features (Alb, monocytes, height, MCV, TP, Lichtiger index, white blood cell count, MCHC, partial Mayo score, and CRP) associated with steroid-free clinical remission at 6 months after starting UST were selected using random forest. The predictive ability of a model using these predictors was evaluated by fivefold cross-validation. Validation of the prediction model with an external cohort showed PPV of 68.8% and NPV of 71.4%. Our study suggested the importance of establishing a drug-specific prediction model.

## Introduction

Ulcerative colitis (UC) is a chronic inflammatory bowel disease (IBD) with unknown etiology that features repeated relapses and remissions^1^. Recently, increased treatment options and improved strategies have allowed more patients to achieve remission and a positive long-term prognosis^2^. An increasing number of molecular-targeted medications, such as calcineurin inhibitors [tacrolimus (TAC)], anti-tumor necrosis factor α (TNFα) antibodies [infliximab (IFX), adalimumab (ADA), and golimumab], anti-α4β7 integrin antibody [vedolizumab (VDZ)], anti-IL12/23p40 antibody [ustekinumab (UST)], and Janus kinase (JAK) inhibitors [tofacitinib (TOF), filgotinib, and upadacitinib], have become available in Japan as therapeutic options for patients with steroid-dependent or steroid-refractory UC. Meanwhile, there is as yet no guide to predict the best molecular-targeted medication for an individual patient. In real-world practice, it is a key clinical challenge for physicians to identify the most effective molecular-targeted therapy for each patient before starting a medication from the perspective of clinical outcomes as well as medical resource use and costs. Molecular-targeted therapies are extremely expensive compared with conventional medications such as 5-aminosalicylic acid (5-ASA), immunomodulatory drugs [e.g., azathioprine (AZA)], and steroids^3^. Predicting the efficacy of molecular-targeted therapies before they are started can prevent the use of ineffective molecular-targeted medications and reduce the socioeconomic burden on patients and healthcare systems.

We previously developed a model for predicting the efficacy of VDZ for UC^4^. Meanwhile, given that molecular-targeted medications have different modes of action, we hypothesized that the model for predicting the therapeutic efficacy for UC can differ for each medication. That is, we considered that our model for predicting the therapeutic efficacy of VDZ (anti-α4β7 integrin antibody) may not be sufficiently predictive for UST (anti-IL12/23p40 antibody) and that a new prediction model specific for UST is needed. Alric et al.^5^ reported that a model for predicting the efficacy of VDZ for Crohn’s disease (CD) patients developed by Dulai et al.^6^ did not work for predicting the efficacy of UST for such patients.

A phase 3 clinical trial on UST for moderate to severe UC demonstrated that the rates of clinical remission, endoscopic improvement, and histo-endoscopic mucosal healing at week 8 were significantly higher in the UST group than in the placebo group; in addition, among all patients who were initially assigned to UST, 77.6% had a clinical response within 16 weeks^7,8^. This effect was observed in patients with or without previous treatment failure with molecular-targeted medications. In addition, Chaparro et al.^9^ analyzed real-world data and reported that the efficacy of UST at week 16 was 53% among moderate to severe cases of UC and that elevated C-reactive protein (CRP) was correlated with failure of remission induction. Meanwhile, specific predictors of the efficacy of UST for UC have yet to be established in other studies^10^. Considering that those clinical studies employed conventional statistical methods, we hypothesized that a machine-learning approach can contribute to exploring predictors of the efficacy of UST for patients with UC. As we conducted a proof-of-concept study on VDZ for UC, the machine-learning approach can provide insights into factors related to clinical outcomes that have not been identified by conventional statistical methodology^4^. In addition, the predictors and a prediction model obtained using the machine-learning approach can be reliable even with a limited sample size^11^. We believe that this is one of the major advantages of the machine-learning approach, allowing researchers to analyze real-world datasets. This characteristic can contribute to developing a prediction model that is feasible and practical in a clinical setting.

In the present study, first, we examined the predictive accuracy of our VDZ prediction model^4^ for UST efficacy among patients with UC. Then, we investigated whether a prediction model specific for UST with higher accuracy can be developed with the machine-learning approach based on real-world clinical data (Table 1).

**Table 1:** Baseline clinical features employed for machine learning.

Background	Treatment history for UC	Concomitant treatment for UC	Clinical activity of UC	Endoscopic activity of UC	Complete blood count	Blood chemistry
Sex (M/F)	5-ASA	5-ASA	Lichtiger index	MES	Red blood cells (× 10^4^/μL)	BUN (mg/dL)
Age (years old)	Azathioprine	Azathioprine	Partial Mayo score		Hemoglobin (g/dL)	Creatinine (mg/dL)
Height (cm)	Prednisolone	Prednisolone			Hematocrit (%)	eGFR (mL/min)
Body weight (kg)	Anti-TNFα agent				MCV (fL)	Total bilirubin (mg/dL)
Body mass index	Tofacitinib				MCH (pg)	AST (IU/L)
UC disease duration (years)	Tacrolimus				MCHC (g/dL)	ALT (IU/L)
UC disease type	CAP				White blood cells (× 10^3^/μL)	GGT (IU/L)
					Neutrophils (%)	Total protein (g/dL)
					Eosinophils (%)	Albumin (g/dL)
					Basophils (%)	Globulin (g/dL)
					Monocytes (%)	CRP (mg/dL)
					Lymphocytes (%)	
					Platelets (× 10^4^/μL)	

*5-ASA* 5-aminosalicylic acid, *TNF* tumor necrosis factor, *CAP* cytapheresis, *UCEIS* ulcerative colitis endoscopic index of severity, *MCV* mean corpuscular volume, *MCH* mean corpuscular hemoglobin, *MCHC* mean corpuscular hemoglobin concentration, *BUN* blood urea nitrogen, *eGFR* estimated glomerular filtration rate, *AST* aspartate aminotransferase, *ALT* alanine aminotransferase, *GGT* gamma-glutamyltranspeptidase, *CRP* C-reactive protein.

## Results

### Training dataset

Baseline characteristics of Cohort 1 are shown in Table 2. Twenty-five patients (13 male and 12 female patients) with active UC were enrolled. Their median age at enrollment was 40 (range 17–85) years, 15 had total colitis and 10 had left-sided colitis, and the mean disease duration was 7.0 (range 0.5–34.6) years. All 25 patients had been treated with 5-ASA, 24 (96.0%) with prednisolone, 20 (80.0%) with anti-TNFα agents, 12 (48.0%) with AZA, and 3 (12.0%) with granulocyte and monocyte apheresis (GMA). No patients had used TAC or TOF before UST. At the initiation of UST, 5-ASA was used concomitantly in 19 (76.0%) patients, while AZA and prednisolone were used in 6 (24.0%) and 4 (16.0%) patients, respectively. Clinical activity at the baseline (upon starting UST) was assessed using LI and partial Mayo (pMayo) score. The median values of LI and pMayo score were 8 (range 5–14) and 6 (3–8), respectively. Regarding endoscopic activity, the median of Mayo endoscopic subscore (MES) at the baseline was 3 (2–3). While clinical activity and blood findings were available for all patients at the baseline, colonoscopy was performed in 21 patients (84.0%). Among the 49 clinical features, five (Ulcerative colitis endoscopic index of severity (UCEIS), UCEIS-V, UCEIS-E, UCEIS-B, and total cholesterol) were excluded from this analysis because data on them were missing in more than 20% of subjects. Thirteen patients (52.0%) achieved steroid-free clinical remission (SFCR) at week 22. No patients withdrew from UST treatment because of adverse events.

**Table 2:** Clinical characteristics of Cohort 1 (training cohort).

Background (N = 25)	
Sex (M/F)	13/12
Age (years old) (median, range)	40 (17–85)
Height (cm)	162.7 ± 1.8
Body weight (kg)	57.2 ± 1.9
Body mass index	21.6 ± 0.6
UC disease duration (years) (median, range)	7.0 (0.5–34.6)
UC disease type (total colitis/left-sided colitis)	15/10
Treatment history for UC	
5-ASA (+/−)	25/0
Azathioprine (+/−)	12/13
Prednisolone (+/−)	24/1
Anti-TNFα agent (+/−)	20/5
Tofacitinib (+/−)	0/25
Tacrolimus (+/−)	0/25
Granulocyte and monocyte apheresis (+/−)	3/22
Concomitant treatment for UC	
5-ASA (+/−)	19/6
Azathioprine (+/−)	6/19
Prednisolone (+/−)	4/21
Clinical activity of UC	
Lichtiger index (median, range)	8 (5–14)
Partial Mayo score (median, range)	6 (3–8)
Endoscopic activity of UC (n = 21)	
Mayo endoscopic subscore (median, range)	3 (2–3)
Complete blood count	
Red blood cells (× 10^4^/μL) (n = 25)	416.7 ± 11.5
Hemoglobin (g/dL) (n = 25)	12.14 ± 0.39
Hematocrit (%) (n = 25)	37.06 ± 1.13
MCV (fL) (n = 25)	89.14 ± 1.71
MCH (pg) (n = 25)	29.22 ± 0.64
MCHC (g/dL) (n = 25)	32.73 ± 0.17
White blood cells (× 10^3^/μL) (n = 25)	7.66 ± 0.44
Neutrophils (%) (n = 24)	64.20 ± 1.93
Eosinophils (%) (n = 24)	3.23 ± 0.51
Basophils (%) (n = 24)	0.81 ± 0.12
Monocytes (%) (n = 24)	9.27 ± 0.63
Lymphocytes (%) (n = 24)	22.24 ± 1.50
Platelets (× 10^4^/μL) (n = 25)	36.44 ± 2.64
Blood chemistry	
BUN (mg/dL) (n = 25)	9.74 ± 0.69
Creatinine (mg/dL) (n = 25)	0.734 ± 0.034
eGFR (mL/min) (n = 25)	85.93 ± 3.81
Total bilirubin (mg/dL) (n = 24)	0.41 ± 0.02
AST (IU/L) (n = 25)	15.7 ± 0.9
ALT (IU/L) (n = 25)	13.8 ± 1.6
GGT (IU/L) (n = 24)	20.3 ± 2.9
Total protein (g/dL) (n = 25)	6.72 ± 0.13
Albumin (g/dL) (n = 25)	3.40 ± 0.13
Globulin (g/dL) (n = 25)	3.31 ± 0.10
CRP (mg/dL) (n = 25)	2.567 ± 0.989

*The results of blood tests are presented as the mean ± SEM.

### Test dataset

Baseline characteristics of Cohort 2 are shown in Table 3. Forty-six patients (25 males and 21 female patients) with active UC were enrolled. Their median age at enrollment was 42 (range 15–85) years, 36 had total colitis, 8 had left-sided colitis, and 2 had proctitis, and the mean disease duration was 5.2 (range 0.2–27.4) years. Overall, 44 (95.7%) patients had been treated with prednisolone, 37 (80.4%) with 5-ASA, 36 (78.3%) with AZA, 24 (52.2%) with anti-TNFα agents, 11 (23.9%) with GMA, 7 (15.2%) with TOF, and 4 (8.7%) with TAC. At the initiation of UST, 5-ASA was used in 37 (80.4%) patients, while AZA and prednisolone were used in 21 (45.7%) and 6 (13.0%) patients, respectively. Clinical activity at the baseline (week 0) was assessed using LI and pMayo score. The median values of LI and pMayo score were 8 (range 5–14) and 5 (2–7), respectively. Regarding endoscopic activity, the median of MES at the baseline was 3 (2–3). While clinical activity and blood findings were available for all patients at the baseline, colonoscopy was performed in 35 patients (76.1%). Twenty-seven patients (58.7%) achieved SFCR at week 22. No patients withdrew from treatment with UST because of adverse events.

**Table 3:** Clinical characteristics of Cohort 2 (testing cohort).

Background (N = 46)	
Sex (M/F)	25/21
Age (years old) (median, range)	42 (15–85)
Height (cm)	163.9 ± 1.2
Body weight (kg)	61.6 ± 2.3
Body mass index	22.8 ± 0.7
UC disease duration (years) (median, range)	5.2 (0.2–27.4)
UC disease type (total colitis/left-sided colitis/proctitis)	36/8/2
Treatment history for UC	
5-ASA (+ / −)	37/9
Azathioprine (+ / −)	36/10
Prednisolone (+ / −)	44/2
Anti-TNFα agent (+ / −)	24/22
Tofacitinib (+ / −)	7/39
Tacrolimus (+ / −)	4/42
Granulocyte and monocyte apheresis (+ / −)	11/35
Concomitant treatment for UC	
5-ASA (+ / −)	37/9
Azathioprine (+ / −)	21/25
Prednisolone (+ / −)	6/40
Clinical activity of UC	
Lichtiger index (median, range)	8 (5–14)
Partial Mayo score (median, range)	5 (2–7)
Endoscopic activity of UC (n = 35)	
Mayo endoscopic subscore (median, range)	3 (2–3)
Complete blood count	
Red blood cells (× 10^4^/μL) (n = 46)	432.3 ± 8.2
Hemoglobin (g/dL) (n = 46)	12.30 ± 0.30
Hematocrit (%) (n = 46)	37.70 ± 0.77
MCV (fL) (n = 46)	86.19 ± 1.57
MCH (pg) (n = 46)	27.91 ± 0.70
MCHC (g/dL) (n = 46)	32.32 ± 0.34
White blood cells (× 10^3^/μL) (n = 46)	7.91 ± 0.45
Neutrophils (%) (n = 46)	65.82 ± 1.63
Eosinophils (%) (n = 46)	3.76 ± 0.53
Basophils (%) (n = 46)	0.63 ± 0.07
Monocytes (%) (n = 46)	6.66 ± 0.37
Lymphocytes (%) (n = 46)	23.08 ± 1.40
Platelets (× 10^4^/μL) (n = 46)	36.68 ± 1.68
Blood chemistry	
BUN (mg/dL) (n = 46)	10.70 ± 0.83
Creatinine (mg/dL) (n = 46)	0.692 ± 0.020
eGFR (mL/min) (n = 45)	89.69 ± 2.57
Total bilirubin (mg/dL) (n = 46)	0.41 ± 0.02
AST (IU/L) (n = 46)	16.5 ± 1.1
ALT (IU/L) (n = 46)	16.1 ± 2.2
GGT (IU/L) (n = 41)	42.6 ± 12.8
Total protein (g/dL) (n = 46)	7.37 ± 0.08
Albumin (g/dL) (n = 46)	3.70 ± 0.08
Globulin (g/dL) (n = 46)	3.67 ± 0.08
CRP (mg/dL) (n = 46)	1.157 ± 0.251

*The results of blood tests are presented as the mean ± SEM.

### Predictive accuracy of the VDZ prediction model for UST treatment

We previously developed a prediction model for VDZ. The positive predictive value (PPV), negative predictive value (NPV), and accuracy for predicting the SFCR at week 22 in the validation cohort in that previous study were 54.5%, 92.3%, and 68.6%, respectively^4^. We applied this prediction model to Cohort 1 in the present study to examine whether it can work for a different medication, UST. We observed that PPV and NPV for SFCR at week 22 were 56.3% and 62.5%, respectively, and overall accuracy was 58.3% (Table 4). These findings underscore the significance of developing a specific, unique model for predicting the clinical efficacy of each medication.

**Table 4:** The accuracy of the VDZ prediction model applied to UST treatment.

	Steroid-free clinical remission at week 22	
	(+)	(−)
Prediction of (+)	9	7
Prediction of (−)	3	5

### Selection of predictive clinical features and development of prediction models

**Figure 1:**
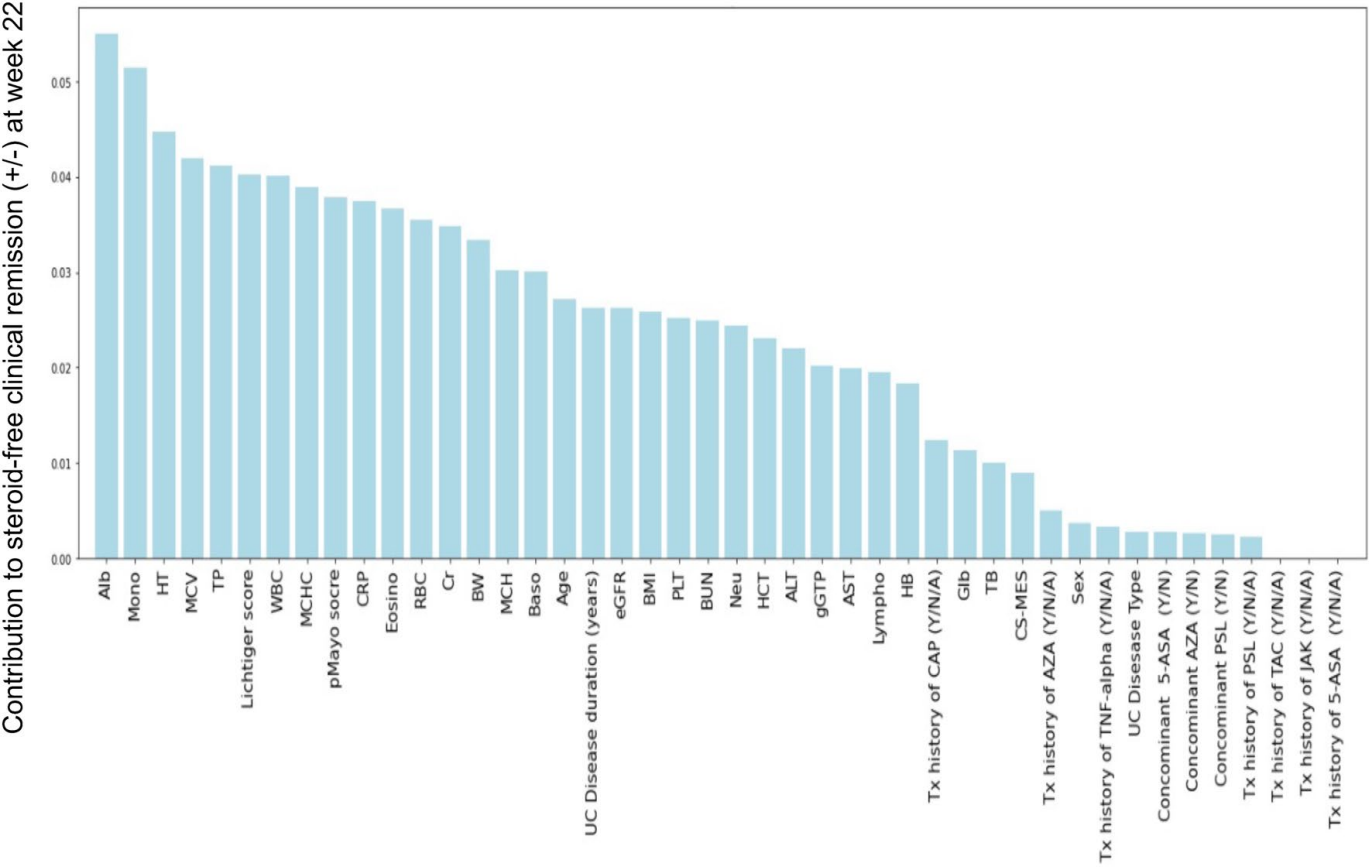
Contributions of 49 clinical features at week 0 to the likelihood of steroid-free clinical remission (SFCR) at week 22. The contribution of each clinical feature at the baseline to SFCR at week 22 was determined using the random forest algorithm. *HT* height, *MCV* mean corpuscular volume, *TP* toral protein, *MCHC* mean corpuscular hemoglobin concentration, *CRP* C-reactive protein, Eosino eosinophil, *pMayo* partial Mayo score, *RBC* red blood cell, *Cr* creatinine, *BW* body weight, *MCH* mean corpuscular hemoglobin, *Baso* basophil, *UC* ulcerative colitis, *BMI* body mass index, *PLT* platelet, *BUN* blood urea nitrogen, *Neu* neutrophil, *HCT* hematocrit, *ALT* alanine aminotransferase, *gGTP* γ-glutamyl transpeptidase, *AST* aspartate aminotransferase, *Lympho* lymphocyte, *HB* hemoglobin, *CAP* cytapheresis, *Glb* globulin, *TB* total bilirubin, *CS-MES* Mayo endoscopic subscore, *AZA* azathioprine, *TNFα* tumor necrosis factor-alpha, *5-ASA* 5-aminosalicylic acid, *PSL* prednisolone, *TAC* tacrolimus, *JAK* Janus kinase inhibitor.

Random forest (RF) using the data of 44 clinical features at the baseline in Cohort 1 was performed, and the contribution of each factor to SFCR at week 22 was determined (Fig. 1). The 10 clinical features with the greatest contributions (positive or negative) to the clinical outcome were serum albumin (Alb) concentration (g/dL), monocyte (Mono) fraction (%), height (HT) (cm), mean corpuscular volume (MCV) (fL), total protein (TP) (g/dL), LI, white blood cell (WBC) count (/μL), mean corpuscular hemoglobin concentration (MCHC) (%), pMayo score, and CRP concentration (mg/dL). These features were employed in the k-nearest neighbor (K-NN) algorithm, logistic regression (L1/L2 regularization), linear/radial basis function (rbf)/polynomial kernel support-vector machine (SVM), and RF to develop a model predictive of the achievement of SFCR at week 22. As shown in Table 5, in Cohort 1 (training cohort), the accuracy was 68.0% to 100%, PPV was 52.0% to 100%, and NPV was 61.1% to 100% in each model.

**Table 5:** The accuracy of predictive models for a training cohort (Cohort 1) developed with the 10 clinical features.

Algorithm	Accuracy	PPV	NPV	Sensitivity	Specificity	AUC
K-NN	0.680	0.857	0.611	0.462	0.917	0.689
Logreg_L1	0.880	0.917	0.846	0.846	0.917	0.881
Logreg_L2	0.840	0.846	0.833	0.846	0.833	0.840
SVM_linear	0.840	0.846	0.833	0.846	0.833	0.840
SVM_poly	0.960	0.929	1.00	1.00	0.917	0.958
SVM_rbf	0.520	0.520	–	1.00	0.00	0.500
RF	1.00	1.00	1.00	1.00	1.00	1.00

*K-NN* k-nearest neighbor algorithm, *Logreg* logistic regression, *SVM* support-vector machine, *rbf* radial basis function, *RF* random forest, *PPV* positive predictive value, *NPV* negative predictive value, *AUC* area under the curve.

### Validation of the prediction model of UST

Next, to validate the predictive ability of each model, we applied the models to Cohort 2 (test cohort). In Cohort 2, the accuracy was 60.9% for the K-NN model, 63.0% for the logistic regression_L1 model, 60.9% for the logistic regression_L2 model, 67.4% for the SVM linear model, 69.6% for the SVM polynomial model, 56.5% for the SVM rbf model, and 67.4% for the RF model (Table 6). The SVM polynomial model demonstrated the highest accuracy with PPV of 68.8% and NPV of 71.4%, followed by the SVM linear model with PPV of 73.9% and NPV of 60.9% and the RF model with PPV of 73.9% and NPV of 60.9%.

**Table 6:** The accuracy of predictive models for a testing cohort (Cohort 2) developed with the 10 clinical features.

Algorithms	Accuracy	PPV	NPV	Sensitivity	Specificity	AUC
K-NN	0.609	0.750	0.533	0.462	0.800	0.631
Logreg_L1	0.630	0.696	0.565	0.615	0.650	0.633
Logreg_L2	0.609	0.682	0.542	0.577	0.650	0.613
SVM_linear	0.674	0.739	0.609	0.654	0.700	0.677
SVM_poly	0.696	0.688	0.714	0.846	0.500	0.673
SVM_rbf	0.565	0.565	–	1.00	0.00	0.5
RF	0.674	0.739	0.609	0.654	0.700	0.677

*K-NN* k-nearest neighbor algorithm, *Logreg* logistic regression, *SVM* support-vector machine, *rbf* radial basis function, *RF* random forest, *PPV* positive predictive value, *NPV* negative predictive value, *AUC* area under the curve.

## Discussion

In the present study, we observed that the VDZ prediction model^4^ did not work sufficiently for UST treatment for UC. This finding supported the notion that a specific prediction model is needed for UST. We identified clinical predictors and developed the UST prediction models using several machine-learning algorithms. We collected clinical information (49 features) at the baseline (week 0, starting UST) of patients treated with UST from our real-world data. The RF algorithm was employed in this study to identify clinical features that contributed positively/negatively to the clinical outcome (i.e., achievement of SFCR at week 22), as in our previous proof-of-concept (POC) study on the efficacy of VDZ for UC^4^. This methodology allows us to examine many clinical features even with limited-size cohorts, which is an advantage in analyzing real-world data. We believe that studies on real-world data can provide crucial insights into optimizing the therapeutic strategy for UC, in addition to conventional clinical trials and ad hoc studies. In clinical trials, to strictly investigate drug efficacy, a wash-out period from the previous treatment is incorporated, and the concomitant use of medications and performance of blood/imaging examinations are controlled. Therefore, the cohorts do not always reflect the real-world clinical setting. In addition, the clinical information collected for trials tends to be limited to clinical features that are widely accepted as being associated with IBD disease activity or commonly accepted as being worth evaluating in IBD clinical research. These points can be limitations of ad hoc studies to explore predictors of treatment efficacy, while the large cohort size and well-documented clinical information can contribute to high-quality analyses. As mentioned in a previous review article^11^, the predictors of the efficacy of VDZ for UC that our POC study^4^ demonstrated on real-world data were compatible with those in another study by Chen et al. using data from the VARSITY^12^ and VISIBLE 1^13^ clinical trials. The numbers of subjects in our training and test cohorts were 34 and 35^4^, while the numbers in VARSITY and VISIBLE 1 were 383 and 160, respectively^14^. Thus, our previous study^4^ proved the concept that an appropriate choice of algorithm makes it possible to draw significant conclusions from real-world data with a limited sample size. Meanwhile, in a clinical study based on real-world data in the clinical setting, it is challenging to control various factors (e.g., sample sizes in training and testing cohorts and the proportions of subjects with vs. without therapeutic efficacy) that could potentially affect the development of prediction models. The robustness against the variability of real-world data could be a selection criterion for prediction models.

Nonetheless, we believe that analyzing the clinical features obtained in a clinical setting before starting UST contributes to identifying predictors and developing a prediction model that can be used in clinical practice, leading to the optimization of IBD treatment in the real world, while we appreciate that experimental factors could be predictors of treatment efficacy and improve the predictive ability of a prediction model^15^. In our study, RF demonstrated the 10 clinical features with the greatest contributions to SFCR at week 22: Alb (g/dL), Mono fraction (%), HT 9 cm), MCV (fL), TP (g/dL), LI, WBC count (/μL), MCHC (%), pMayo score, and CRP concentration (mg/dL). The finding that indexes of nutrition status (Alb and TP), indexes related to anemia (e.g., bleeding or iron deficiency) (MCV and MCHC), indexes of clinical activity (LI and pMayo score), and indexes of inflammation or concomitant infection (WBC and CRP) are included as predictors may support our clinical experience that UST is effective for patients with moderate UC rather than severe UC, or that patients with a better general condition tend to be more responsive to treatment. This speculation also seems reasonable given that, in our previous report on the efficacy of VDZ for UC, pMayo score, MCH, body mass index, blood urea nitrogen, CRP, and total cholesterol were included among the top 10 contributors to SFCR at week 22^4^. Meanwhile, it is interesting that Mono fraction (%) were identified as predictors by RF. UST is an antibody against IL-12/23p40 and is considered to block the pathways related to IL-12 and IL-23^7^. IL-23 in particular plays an important role in the pathogenesis of IBD, and anti-IL-23 antibodies, such as risankitzumab^16^, guselkumab^17^, and mirikizumab^18^, are now used for IBD. Intestinal macrophages contribute to chronic intestinal inflammation via IL-23 production^19^. Given that macrophages are classified within the category of monocytes, the Mono fraction in the blood may reflect the activity of intestinal macrophages. Meanwhile, our previous study identified the fraction of lymphocytes in the blood as a predictor of the efficacy of VDZ for UC. VDZ is an anti-α4β7 integrin antibody. The α4β7 integrin is expressed mainly on lymphocytes and binds to adhesion molecules on vascular endothelial cells so that the lymphocytes migrate to the gastrointestinal mucosa. That is, VDZ blocks lymphocyte migration and suppresses the inflammation in the intestinal mucosa^4^. These findings raised the idea that our previous observation reflects the contribution of lymphocytes to intestinal inflammation. Considering prediction models for UST and VDZ included the fraction of monocytes and lymphocytes, respectively, the difference in drug action mechanisms between VDZ and UST could be a reason why the prediction model for VDZ does not work sufficiently for UST. Furthermore, a recent systematic review suggested that higher eosinophil levels in colonic tissue and/or blood are associated with increased disease activity and poorer response to therapy in UC^20^. Eosino fraction (%) in blood, the 11th contributor in our RF (almost the same contribution as CRP), may be related to the clinical significance of eosinophils. It is noteworthy that, also in CD, monocytes and eosinophils at the baseline were identified as among the top 10 contributors to UST efficacy^21^.

After determining the contribution of clinical features to SFCR at week 22, we compared various algorithms to develop a prediction model using the top 10 predictors and observed that the SVM polynomial model, the SVM linear model, and the RF model seem promising. Considering the differences in predictive accuracy between the training and testing cohorts, although it is common that predictive accuracy is observed better in the training cohort compared to the testing cohort, we cannot exclude the possibility that overfitting occurred in the training cohort in the study. The present study, together with our previous report^4^, supports the notions that a prediction model specific to each medication should be used and that a machine-learning approach is useful for several medications. Meanwhile, we acknowledge certain challenges and limitations associated with this study. First, in the present study, we employed 44 clinical features that were retrospectively available and compatible with our previous study^4^ in this study and, considering the calculated contributions of each feature, decided to select the top 10 contributors out of 44 features as predictors for a prediction model. In this study design, we cannot exclude the possibility that clinical features not included in the 44 clinical features impact the UST efficacy. However, since we collected clinical features as many as possible retrospectively, given the feasibility in clinical practice, these clinical features can be considered to contain almost all features available in the clinical setting. Meanwhile, while the use of fewer variables can be more convenient in clinical practice, the “appropriate” number of clinical features to be input into a model remains an important issue. Second, determining the “best” machine-learning algorithm(s) for a prediction model is a key challenge. We investigated 7 major algorithms in the present study but there remains a possibility that other algorithms have better predictive ability. Our findings demonstrated that the optimal algorithm for a prediction model can differ between medications. We also consider that not a single “best” algorithm but the combination of multiple algorithms may contribute to improving the predictive accuracy. It is challenging to determine what algorithm(s) should be preferentially examined for each medication and how multiple algorithms can be combined. Third, larger training and test cohorts could be an advantage for identifying predictors and developing a more accurate prediction model, although the machine-learning approach has merit in the analysis with limited-size cohorts. Also, cohorts with further long follow-up periods would contribute to developing a prediction model for the best practice in UC treatment. While our prediction model was developed to predict the SFCR at week 22, the prediction of the longer-term prognosis including loss of response is another crucial clinical challenge. Also, cohorts with further long follow-up periods would contribute to developing a prediction model for the best practice in UC treatment. While our prediction model was developed to predict the SFCR at week 22, the prediction of the longer-term prognosis including loss of response is another crucial clinical challenge. As future perspectives, it would be interesting to input additional feasible/practical clinical information, such as biomarkers and histological findings, into the machine-learning process. These features have been reported to be related to the prognosis of patients with IBD^22,23^ and may contribute to predicting treatment efficacy. In addition, expanding the training and test cohorts to other facilities and to other medications is crucial for developing accurate models and predicting the best medication for each patient, which should eventually lead to personalized medicine in IBD. The assessment of the predictive ability of the prediction model in prospective studies could provide insights for future clinical applications. It would be also interesting to apply our machine-learning approach to international cohorts and investigate if there are differences between regions. While we employ clinical features for developing a prediction tool in a clinical setting, we believe that inputting various meta-data including cutting-edge research findings not clinically applied yet (e.g., cytokine profiles, mucosal gene expression, and the gut microbiome) for machine learning and interpreting the physiological significance of the computed results for the clinical outcome would be an interesting way of discovering novel factors involved in the treatment efficacy and pathogenesis of IBD. Also, these various features would contribute to developing a computational model of IBD pathogenesis. Rogers et al. reported the potential of a dynamic quantitative systems pharmacology model for considering immune systems in IBD^24^. Although modeling IBD should be highly complicated and a large number of samples needed for developing a model would be a restriction, a simulation model of immune networks in IBD could provide an opportunity to consider therapeutic strategies based on IBD pathoetiology.

In conclusion, we determined the contribution of clinical features at the baseline (week 0) to the achievement of SFCR at week 22 in patients treated with UST for UC, developed a model for predicting SFCR at week 22 with UST for UC, and validated the predictive ability of this model. The methodology and findings in this study could be applied to other medications and diseases.

## Methods

### Study setting and outcomes

This work involved a multi-center retrospective study aimed at developing a model for predicting the efficacy of UST for patients with UC. The diagnosis of UC was confirmed using the clinical practice guidelines for IBD of The Japanese Society of Gastroenterology^1^. UST treatment for the induction of remission was defined as when UST was started for active UC [Lichtiger index^25^ (LI) ≥ 5]. Patients who started UST as induction treatment, underwent blood testing at baseline (week 0), and could be tracked for assessment of clinical activity at 22 weeks after starting UST (week 22) were enrolled in this study. Patients who started UST between June 2020 and July 2021 at Kyorin University Hospital (Tokyo, Japan) were defined as the training cohort (Cohort 1), while those who started UST between June 2020 and February 2022 at Toho University Sakura Medical Center (Chiba, Japan) were defined as the test cohort (Cohort 2). SFCR at week 22 was evaluated as the clinical outcome. SFCR was defined as an LI of 4 or lower. Patients who terminated UST treatment or needed surgery because of insufficient control of UC inflammation before week 22 were regarded as not achieving clinical remission.

### Clinical information

The clinical features retrospectively obtained from medical records at both facilities were designated to be compatible with those in our previous report on developing a prediction tool for VDZ treatment^4^. Clinical information [age, sex, height, body weight, body mass index (BMI), UC disease type, UC disease duration, treatment history for UC, pMayo score^26^, LI], endoscopic activity [MES^26^, UCEIS^27^], and 25 blood test findings (Table 1) were collected from medical charts at the time of starting UST. Findings of colonoscopy (endoscopic activity assessment) performed within 3 months before starting UST were collected as the baseline endoscopic findings.

### Machine learning procedure and statistical analysis

Continuous variables are expressed as median and interquartile range (IQR) or mean and standard deviation (SD). Missing values were imputed with the average value and the mode value for numerical data and categorical data, respectively. However, if data were missing for more than 20% of subjects in an item, this item was not included in the analysis. The standardized values of Cohort 1 were used for the feature selector composed of RF. RF was employed to identify which feature contributed to the prediction and develop a highly accurate prediction model in the present study. In training, the RF algorithm creates multiple trees, and each tree is trained on the bootstrapped samples of the training data. Since the number of patients was limited in this study, it was not possible to accurately calculate the contribution of a feature in a single training process. Therefore, the feature selector was initially set as zero, and the training and importance calculation were repeated 50 times. The contribution of each feature (49 clinical features, Table 1) to SFCR at week 22 was obtained by calculating the average value. When training the RF, the hyperparameters (number of trees and maximum depth of the tree) were automatically optimized via grid search and cross-validation. Grid search is a method for obtaining optimal hyperparameters in an algorithm. This methodology performs a complete search over a given subset of the hyperparameter space of the training algorithm. The best hyperparameters are estimated according to the evaluation score of the validation data. In this study, we used accuracy as the evaluation score for grid search. The candidate hyperparameters for RF were the number of trees (10, 20, 30, 40, 50, 60, 70, 80, 90) and the maximum depth of the trees (1, 2, 3, 4, 5, 6, 7, 8, 9). Cross-validation is a resampling procedure for evaluating machine-learning models on a limited data sample. The general procedures are as follows: (1) split the dataset into k groups; (2) for each group, (i) select a group as a validation dataset, (ii) use the remaining groups (“k − 1” groups) as a training dataset, and (iii) fit a model on the training set and evaluate it on the validation set; and (3) calculate an average of k evaluation score. In (1), stratified K-fold was used to ensure that there is no variation in class proportions among groups. The final prediction results were obtained from the mode of predictions obtained from individual decision trees. The feature importance is determined according to the extent a decision tree node using each feature can reduce impurity across all trees in the forest. Next, logistic regression, the K-NN algorithm, linear/radial basis function (rbf)/polynomial kernel SVM, and RF were each used to develop prediction models in this study. We used grid search and cross-validation for training as well as a feature selector for the predictive models. We inputted 10 clinical features at week 0 that were selected as features with high contributions based on RF findings to predict the achievement of SFCR or lack thereof at week 22. The predictive accuracy of the model was assessed using the data of Cohort 2. We performed the machine learning in Python and used the scikit-learn package.

### Ethical considerations

This study was conducted in accordance with the Declaration of Helsinki. This study was approved by the Faculty of Medicine Research Ethics Committee, Kyorin University (Approval Number 1814) and the Ethics Committee of Toho University Sakura Medical Center (Approval Number S21082). This study used data that had already been recorded and the ethics committees (the Faculty of Medicine Research Ethics Committee, Kyorin University and the Ethics Committee of Toho University Sakura Medical Center) waived the need to obtain informed consent.

## Data Availability

The data underlying this article are available from the corresponding author upon reasonable request.

## Abbreviations

5-ASA: 5-Aminosalicylic acid
ADA: Adalimumab
Alb: Albumin
AZA: Azathioprine
BMI: Body mass index
BUN: Blood urea nitrogen
CD: Crohn’s disease
CRP: C-reactive protein
Eosino: Eosinophil
GMA: Granulocyte and monocyte apheresis
IBD: Inflammatory bowel disease
IFX: Infliximab
JAK: Janus kinase
K-NN: K-nearest neighbor
LI: Lichtiger index
Lympho: Lymphocyte
MCH: Mean corpuscular hemoglobin
MCHC: Mean corpuscular hemoglobin concentration
MCV: Mean corpuscular volume
MES: Mayo endoscopic subscore
NPV: Negative predictive value
pMayo score: Partial Mayo score
PPV: Positive predictive value
PSL: Prednisolone
POC: Proof-of-concept
RF: Random forest
rbf: Radial basis function
SFCR: Steroid-free clinical remission
SD: Standard deviation
SVM: Support-vector machine
TAC: Tacrolimus
TCho: Total cholesterol
TNF: Tumor necrosis factor
TOF: Tofacitinib
TP: Total protein
UC: Ulcerative colitis
UCEIS: Ulcerative colitis endoscopic index of severity
UST: Ustekinumab
VDZ: Vedolizumab
WBC: White blood cell

## References

[1] Nakase H (2021). Evidence-based clinical practice guidelines for inflammatory bowel disease 2020. J. Gastroenterol..

[2] Fumery M (2018). Natural history of adult ulcerative colitis in population-based cohorts: A systematic review. Clin. Gastroenterol. Hepatol..

[3] Park KT (2020). The cost of inflammatory bowel disease: An initiative from the Crohn’s & Colitis foundation. Inflamm. Bowel Dis..

[4] Miyoshi J (2021). Machine learning using clinical data at baseline predicts the efficacy of vedolizumab at week 22 in patients with ulcerative colitis. Sci. Rep..

[5] Alric H (2022). Vedolizumab clinical decision support tool predicts efficacy of vedolizumab but not ustekinumab in refractory crohn’s disease. Inflamm. Bowel Dis..

[6] Dulai PS (2018). Development and validation of a scoring system to predict outcomes of vedolizumab treatment in patients with Crohn’s disease. Gastroenterology.

[7] Sands BE (2019). Ustekinumab as induction and maintenance therapy for ulcerative colitis. N. Engl. J. Med..

[8] Hisamatsu T (2021). Efficacy and safety of ustekinumab in East Asian patients with moderately to severely active ulcerative colitis: A subpopulation analysis of global phase 3 induction and maintenance studies (UNIFI). Intest. Res..

[9] Chaparro M (2021). Effectiveness and safety of ustekinumab in ulcerative colitis: Real-world evidence from the ENEIDA registry. J. Crohns Colitis.

[10] Gisbert JP, Chaparro M (2020). Predictors of primary response to biologic treatment [Anti-TNF, vedolizumab, and ustekinumab] in patients with inflammatory bowel disease: From basic science to clinical practice. J. Crohns Colitis.

[11] Pinton P (2022). Prediction of vedolizumab treatment outcomes by machine learning. J. Biopharm. Stat..

[12] Sands BE (2019). Vedolizumab versus adalimumab for moderate-to-severe ulcerative colitis. N. Engl. J. Med..

[13] Sandborn WJ (2020). Efficacy and safety of vedolizumab subcutaneous formulation in a randomized trial of patients with ulcerative colitis. Gastroenterology.

[14] Chen J, Girard M, Wang S, Kisfalvi K, Lirio R (2022). Using supervised machine learning approach to predict treatment outcomes of vedolizumab in ulcerative colitis patients. J. Biopharm. Stat..

[15] Verstockt B (2020). Expression levels of 4 genes in colon tissue might be used to predict which patients will enter endoscopic remission after vedolizumab therapy for inflammatory bowel diseases. Clin. Gastroenterol. Hepatol..

[16] D'Haens G (2022). Risankizumab as induction therapy for Crohn’s disease: Results from the phase 3 ADVANCE and MOTIVATE induction trials. Lancet.

[17] Sandborn WJ (2022). Guselkumab for the treatment of Crohn’s disease: Induction results from the phase 2 GALAXI-1 study. Gastroenterology.

[18] Sandborn WJ (2020). Efficacy and safety of mirikizumab in a randomized phase 2 study of patients with ulcerative colitis. Gastroenterology.

[19] Kamada N (2008). Unique CD14 intestinal macrophages contribute to the pathogenesis of Crohn disease via IL-23/IFN-gamma axis. J. Clin. Invest..

[20] Mookhoek A (2022). The clinical significance of eosinophils in ulcerative colitis: A systematic review. J. Crohns Colitis.

[21] Waljee AK (2019). Development and validation of machine learning models in prediction of remission in patients with moderate to severe crohn disease. JAMA Netw. Open.

[22] Theede K (2016). Fecal calprotectin predicts relapse and histological mucosal healing in ulcerative colitis. Inflamm. Bowel Dis..

[23] Ozaki R (2018). Histological risk factors to predict clinical relapse in ulcerative colitis with endoscopically normal mucosa. J. Crohns Colitis.

[24] Rogers KV, Martin SW, Bhattacharya I, Singh RSP, Nayak S (2021). A dynamic quantitative systems pharmacology model of inflammatory bowel disease: Part 1 - model framework. Clin. Transl. Sci..

[25] Lichtiger S (1994). Cyclosporine in severe ulcerative colitis refractory to steroid therapy. N. Engl. J. Med..

[26] Schroeder KW, Tremaine WJ, Ilstrup DM (1987). Coated oral 5-aminosalicylic acid therapy for mildly to moderately active ulcerative colitis. A randomized study. N. Engl. J. Med..

[27] Mohammed Vashist N (2018). Endoscopic scoring indices for evaluation of disease activity in ulcerative colitis. Cochrane Database Syst. Rev..

